# Detection of *Bartonella henselae* DNA in *Triatoma sordida* collected in peridomiciliary environments

**DOI:** 10.1016/j.bjid.2024.103875

**Published:** 2024-09-28

**Authors:** Luciene Silva dos Santos, Jader Oliveira, Vagner José Mendonça, João Aristeu Rosa, Alexandre Seiji Maekawa, Maurício Lilioso, Dayane Pires da Silva, Carlos Eduardo Almeida, Paulo Eduardo Neves Ferreira Velho, Marina Rovani Drummond

**Affiliations:** a Universidade de Campinas (UNICAMP), Faculdade de Ciências Médicas, Laboratório de Pesquisa Aplicada em Dermatologia e Infecção por Bartonella, Campinas, SP, Brazil; b Universidade de São Paulo (USP), Faculdade de Saúde Pública, Laboratório de Entomologia em Saúde Pública, São Paulo, SP, Brasil; c Universidade Federal do Piauí, Departamento de Parasitologia e Microbiologia, Teresina, Piauí, Brasil; d Universidade Estadual Paulista (Unesp), Faculdade de Ciências Farmacêuticas, Laboratório de Parasitologia, Araraquara, SP, Brasil; e Faculty of Medicine – Endocrinology, Memorial University of Newfoundland, St. John's, Newfoundland and Labrador, Canada; f Universidade Estadual de Campinas (UNICAMP), Instituto de Biologia, Departamento de Biologia Animal - Parasitologia, Campinas, SP, Brasil; g Universidade de Campinas (UNICAMP), Instituto de Biologia, Programa de Pós-Graduação em Genética e Biologia Molecular, Campinas, SP, Brazil; h Universidade Federal do Rio de Janeiro (UFRJ), Instituto de Biologia, Laboratório de Entomologia, Rio de Janeiro, RJ, Brazil; i Universidade de Campinas (UNICAMP), Faculdade de Ciências Médicas, Departamento de Medicina, Divisão de Dermatologia, Campinas, SP, Brazil

**Keywords:** *Bartonella henselae*, Chagas disease, Endocarditis, *Trypanosoma cruzi*

## Abstract

Bartonelloses represent a group of potentially fatal diseases associated with various clinical manifestations including endocarditis. Caused by bacteria belonging to the genus *Bartonella*, these microorganisms have a remarkable ability to infect mammals, and their transmission is commonly associated with hematophagous vectors such as fleas, lice, mosquitoes, and ticks. The aim of this study was to evaluate the occurrence of *Bartonella* sp. DNA in 81 triatomines of the species *Triatoma sordida* collected in the field in peri‑domiciliary areas of the Brazilian city of Seabra, located in the state of Bahia. Nested PCR was conducted targeting the *ftsZ* gene and real-time PCR targeting the *gltA* gene, both representing specific reactions for *Bartonella henselae*. Additionally, conventional PCR targeting kDNA was employed to evaluate the presence of *Trypanosoma cruzi.* Of the samples tested, 23/81 (28.39 %) bugs showed positive PCR for *B. henselae*. No sample showed positive PCR for *T. cruzi*. The high prevalence of triatomines with a positive PCR for *B. henselae* emphasizes the close relationship between these insects and the bacteria, indicating the need for further studies to investigate the vectorial potential of these kissing bugs.

## Introduction

Among the zoonoses of interest in public health is Chagas Disease (CD), considered neglected by the World Health Organization (WHO). It is estimated that this disease affects around 6‒7 million people worldwide, and its underreporting emerges as a challenge since this disease has notable chances of cure when treated early.^1^ A study evaluating mortality caused by neglected diseases in Brazil between 2000‒2011 showed that CD was responsible for 76.7 % of all deaths.^2^

The agent of CD, *Trypanosoma cruzi*, is known to have several triatomines species associated with its transmission, in addition to demonstrating a wide variety of mammalian hosts. Among these hosts, many are carriers of other pathogens that also employ hematophagous arthropods as vectors.^3^, ^4^, ^5^

One such group of pathogens includes the genus *Bartonella*, comprising over 40 species of gram-negative bacteria.^6^ These bacteria are primarily transmitted by vectors like fleas, sandflies, and lice, with potential vectors including bed bugs and ticks.^7^, ^8^, ^9^ *Bartonella* species prefer infecting mammalian erythrocytes and endothelial cells and have a broad host range, including various sylvatic and domestic animals.^10^^,^^11^Globally, *Bartonella henselae* is the most common species causing clinical manifestations in humans, dogs, and cats.^12^, ^13^, ^14^

The ability of *Bartonella* species to infect a wide variety of mammals, including several that serve as reservoirs for *T. cruzi*, raises the possibility of triatomines also being potential vectors of these bacteria, as observed in other research, given these close ecological relationships.^5^ Recently, in southern China, a study detected DNA from *Bartonella* species in 36.4 % (8/22) of *Triatoma rubrofasciata* analyzed.^15^ This species of triatomines has a cosmopolitan distribution, emerged outside Latin America and plays a global role in the transmission of *Trypanosoma conorhini*, a parasite with no reports of infection in humans. In the Americas, this insect is considered a secondary vector of *T. cruzi*.^15^^,^^16^ Previously, a study in French Guiana had reported a candidate species *Bartonella rondonienses* in 13/23 (56.5 %) triatomines of the species *Eratyrus mucronatu*s.^9^ Although considered a species with wild habits, there is an adaptation of *E. mucronatus* to the destruction of its habitat, approaching areas inhabited by humans.^17^ This species has already been associated with occasional transmission of CD in Bolivia, and *T. cruzi* has already been detected in it in Brazil.^18^^,^^19^

A study conducted with 73 patients with chagasic cardiomyopathy detected *Bartonella* sp. DNA in 34 of them (46.6 %).^20^ Compared to the control group, these patients had 40 times more chances of presenting the infection. In the same study, Argentine patients seroreactive for CD without clinical disease (therefore with indeterminate CD) also showed a high prevalence of 11/32 (34.4 %) *Bartonella* sp. infection, a risk 2.5 times higher than patients with chagasic cardiomyopathy from the same country.

*Triatoma sordida* is the most collected triatomine species in Brazil in terms of absolute numbers, by entomological surveillance, being widely adapted to the peridomicile.^21^ Like *Triatoma brasiliensis*, this species is considered a secondary vector of *T. cruzi*, a significant epidemiological concern in Brazil.^22^ This species is naturally found under tree bark and also in bird nests.^23^ In the peridomicile, *T. sordida* is commonly found in chicken coops and under pieces of wood.^24^^,^^25^ And even though associated with birds, individuals infected with *T. cruzi* are found in these environments, posing a risk of CD transmission, especially for residents of rural areas.

Based on these findings, the present study was conducted with the purpose of investigating the co-detection of *B. henselae* DNA and its possible coinfection with *T. cruzi* in Brazilian triatomines collected in the peridomicile.

## Materials and methods

In accordance with institutional guidelines and applicable animal ethics regulations, research involving insects and other invertebrates is exempt from animal ethics committee approval, as these organisms are not subject to oversight under current ethics review policies.

**Fig. 1:**
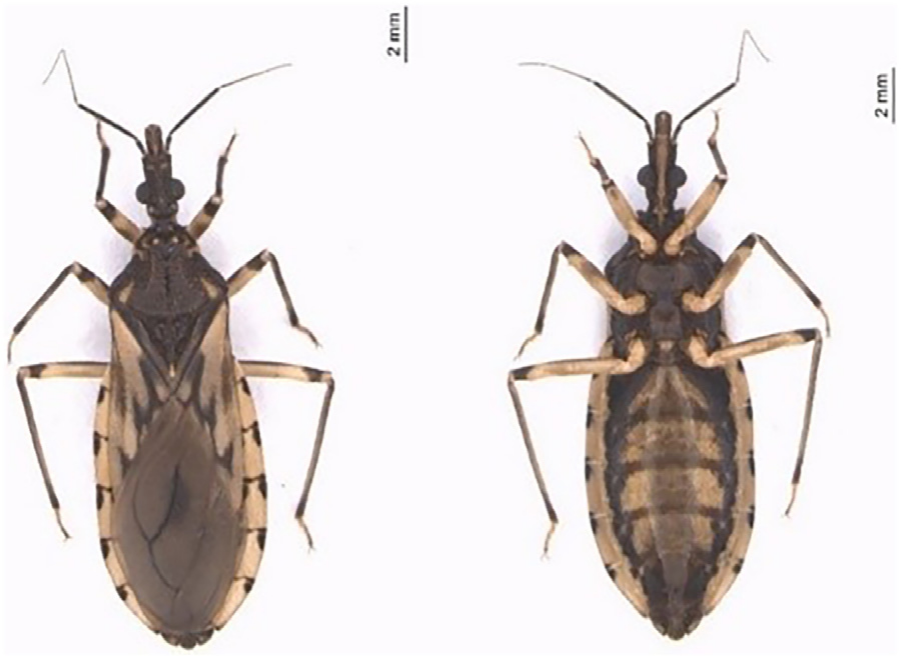
Dorsal and ventral view of an adult female *Triatoma sordida*. Photos from the Image Bank of the Triatominae Collection ‒ Faculty of Pharmaceutical Sciences ‒ São Paulo State University, Araraquara campus.^26^

To analyze the occurrence of *B. henselae* and the potential co-detection with *T. cruzi* in triatomines collected in the field, the presence of DNA from these agents in bugs of the species *T. sordida* (Fig. 1) was analyzed.

**Fig. 2:**
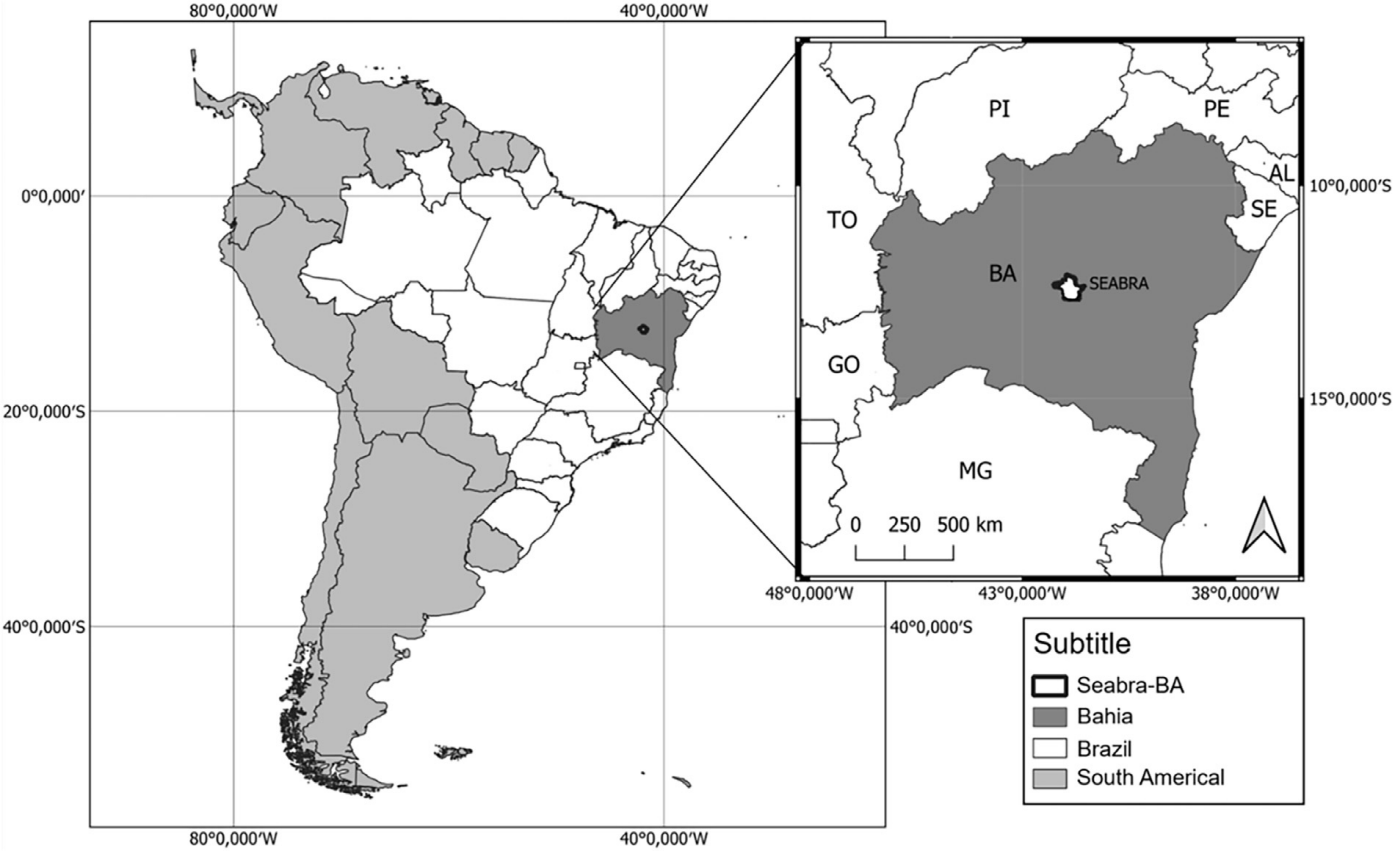
In the image, a map of Brazil highlighting the state of Bahia. In closer view, the location of the city of Seabra in this state is observed.

The triatomines analyzed in this study were obtained in March 2013 in peridomestic regions in the city of Seabra (latitude 12°25′3.03″S and longitude 41°46′9.21″W) in the state of Bahia, Brazil (Fig. 2). Insect collection was carried out manually, and subsequently, they were stored in 70 % alcohol.

### Sample preparation

Surface decontamination of the triatomines was performed by two immersions, with agitation, in ethanol (70 %), each lasting 10 min. Subsequently, the insects were immersed in sterile PBS for 5 min and then dried in a laminar flow. Using a sterile scalpel, wings, legs, and antennae were removed, and the insects were cut sagittally. One-half was randomly selected for analysis, while the other part was stored. Each half designated for analysis was individually deposited into 1.5 mL microtubes and macerated with a sterile pestle using liquid nitrogen.

### DNA extraction

DNA extraction was performed using the commercial *QIAamp* DNA *Mini Kit* (Qiagen®, Hilden, Germany) following the manufacturer's protocol.

### Molecular analyses

In all samples, a PCR reaction was performed for a specific endogenous gene of the Triatominae subfamily targeting the Cytochrome Oxidase I (COI) fragment.^27^ This procedure aimed to evaluate both the quality of the extracted DNA and the absence of inhibitors in the reaction.

Subsequently, the samples were subjected to nested PCR analysis targeting the *ftsZ* gene specific to *B. henselae* and real-time PCR directed at the *gltA* gene, also specific to the same species of *Bartonella*.

Two types of controls were added to all reactions: one with only reagents of each reaction to ensure no contamination between the reagents and another with serial dilutions of *B. henselae* DNA to determine the sensitivity and limit of detection of each reaction. To prevent cross-contamination, the *B. henselae* DNA used as a control was synthetically prepared in such a way that its fragment was distinct from the expected fragment by the DNA band of wild *B. henselae*, thus easily identifiable and avoiding false positives resulting from contamination, as described.^28^

All samples were analyzed by conventional PCR for *T. cruzi* identification. The first PCR reaction was performed using the primers 121 (Forward) AAATAATGTACGGG (T/G) GAGATGCATGA and 122 (Reverse) GGTTCGATTGGGGTTGGTGAATATA, previously described by Sturm et al. and Wincker et al., which amplify fragments of the kinetoplast DNA (kDNA) of *T. cruzi* and *Trypanosoma rangeli*, a trypanosomatid also found in triatomines but not pathogenic to humans and other vertebrates.^29^^,^^30^ The PCR reaction and cycling conditions of this first reaction followed the steps of Valença-Barbosa et al. ^31^ The second reaction was performed with the primers TcH2AF and TcH2AR, isolating a fragment present in the non-coding 3′ region of the 1.2 kb histone H2A coding unit specifically from *T. cruzi*.^32^ The protocol used for this reaction was the same as that used by Lilioso M et al. ^33^

The PCR products were applied to a 2 % agarose gel and visualized with GelRed staining (Biotium Inc., Hayward, CA, USA). The absence of amplification products on the gel, specifically at 330 base pairs for the 121/122 primers and 234 base pairs for the TcH2AF/TcH2AR primers, was interpreted as indicative of no *T. cruzi* infection in the samples. All PCRs were performed with three positive controls for *T. cruzi*, including one DNA control extracted from a *T. cruzi* culture (TcI), and the other two from DNA extracted from the abdominal contents of *Triatoma brasiliensis* genotyped previously. Additionally, the negative controls used were NTC (non-template control) and a *T. rangeli* DNA.

## Results

Eighty-one triatomines were analyzed. All samples showed amplification of the endogenous gene.

No sample showed DNA amplification for *T. cruzi*.

**Fig. 3:**
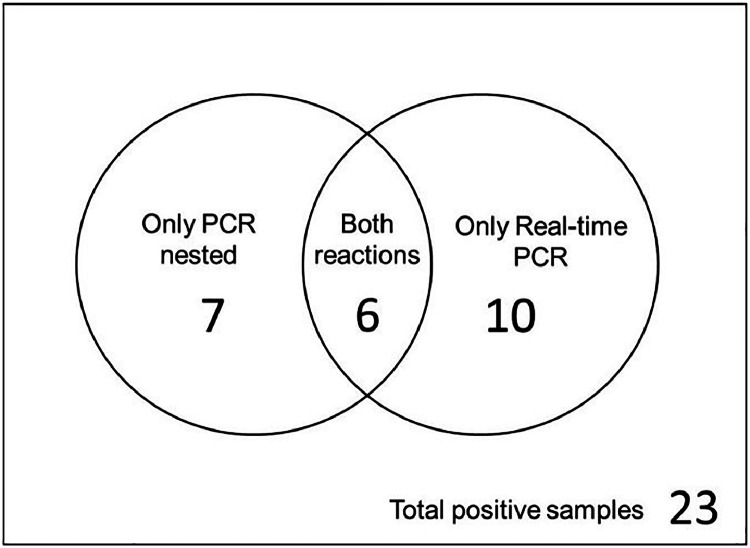
Venn diagrams representing positive *Bartonella* sp. ‒ PCR results showing how many samples were amplified in each reaction and those detected in more than one PCR.

*B. henselae* DNA was detected in 23 out of 81 individuals (28.39 %) in at least one of the two species-specific reactions for *B. henselae*, with 13/23 in nested PCR (56.52 %) and 16/23 in real-time PCR (69.56 %). In six samples, both reactions (26.08 %) detected the bacterial DNA. The results can be better observed in the Venn diagram (Fig. 3).

Of the 23 samples that resulted in DNA amplification, 9 (39.13 %) were subjected to sequencing, with seven amplicons obtained from nested PCR and four from real-time PCR. Two samples had their amplicons recovered from both reactions. All samples showed 100 % similarity with *B. henselae*.

## Discussion

**Fig. 4:**
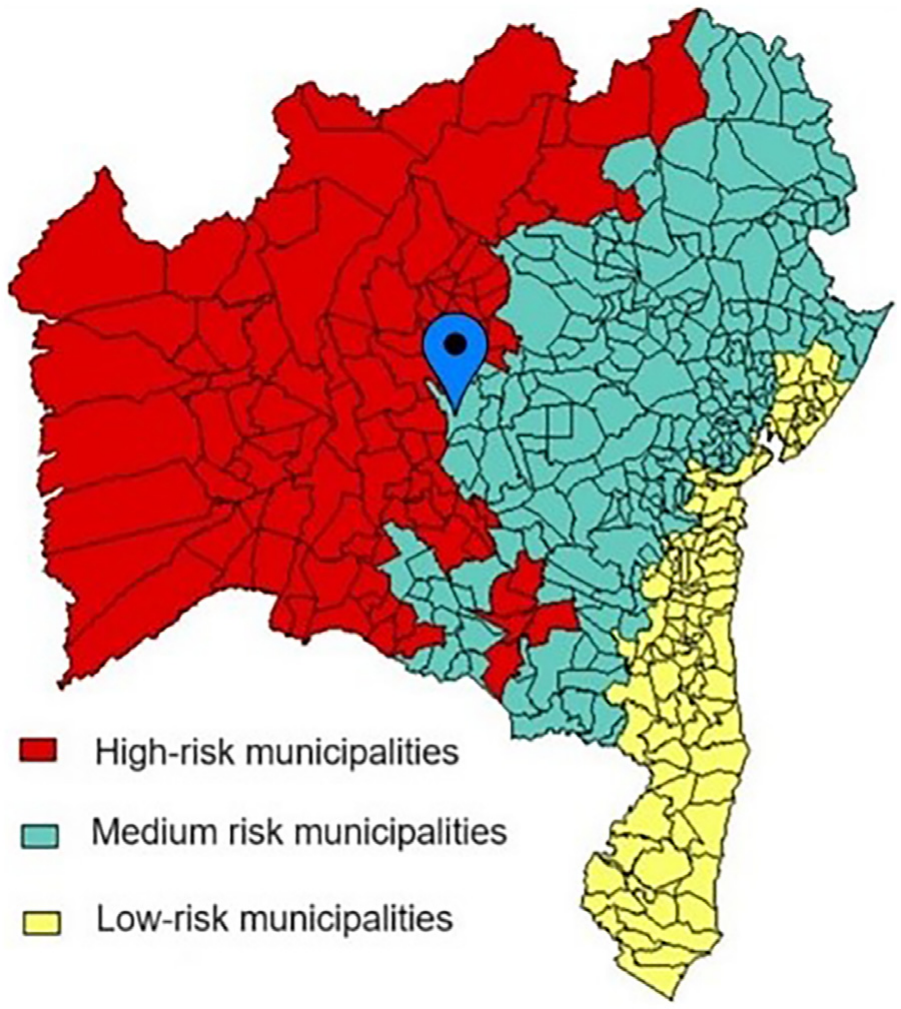
Adapted map with the location of the city of Seabra within the distribution of cities with a medium degree of risk of Chagas disease transmission. Bahia, 2012.^34^

The study found a high prevalence of *Bartonell*a sp. DNA detection in *T. sordida* collected in the peridomicile in an area with medium risk of vector-borne transmission of CD despite the low parasitic infection found in *T. sordida* from the same region (Fig. 4).^34^

The detection of the bacterial DNA is not sufficient to confirm the vectorial capacity of triatomines to infect hosts through biting or feces.^35^ The genetic material could be into the blood freshly sucked from other animals, potential reservoirs of *Bartonella* spp.^36^ Previous studies evaluated the ability of *Bartonella quintana* to multiply in the intestine of bed bugs and demonstrated its viability in the feces of these insects.^7^ A similar study should be done with triatomine species.

The results of the study with patients with chagasic cardiomyopathy and indeterminate disease revealed a high prevalence of *Bartonella* sp. infection in these individuals.^20^ This observation suggests the possibility of the bacterial transmission by the same vector or by other hematophagous arthropods sharing the same environment.

Furthermore, if confirmed the intestinal multiplication of *Bartonella* sp. in triatomines, it will be necessary to evaluate whether the transmission of the bacterium occurs during the blood meal or, as with *T. cruzi*, from contact of the skin or mucous membranes with the feces of the arthropods. Transmission of *Bartonella* spp. through feces is the most likely, considering that in cats transmission between infected and uninfected animals does not occur without contact with feces from infected fleas.^37^^,^^38^ It is also important to observe if *Bartonella* spp. has the ability to multiply in the salivary glands of triatomines.

Given that *B. henselae* is the most frequent cause of disease in humans, species-specific reactions were used for this bacterium.^39^ Genus-specific PCRs should also be opportunistically performed in *Triatoma sordida* and other triatomine species, since *Bartonella* DNA has already been found in *E. mucronatus* and *T. rubrofasciata*.^9^^,^^15^

Based on the results of this study, further research is needed to confirm the vectorial competence of triatomines in acting as vectors of *Bartonella* spp., as the limitation of this study was finding DNA of these pathogens and not their viability. New research is also needed to investigate the diversity of *Bartonella* spp. present in these insects, as the analyses performed only targeted *B. henselae*. Likewise, it is necessary to verify the prevalence of these pathogens in other triatomine species. Since little is known about the ways that *Bartonella* spp. are maintained in natural environments, it is also crucial to verify if the cycle of these bacteria can be maintained through the entomophagy of hematophagous insects by potential vertebrate reservoirs.

## Conclusion

The results of this study reveal high detection of *B. henselae* DNA in triatomines of the species *T. sordida* collected in peridomiciliary areas of the city of Seabra, Bahia. These findings have significant implications for the epidemiology of both diseases.

## Conflicts of interest

The authors declare no conflicts of interest.

## Data Availability

Data generated or analyzed during this study are available from the corresponding author upon reasonable request.
